# Mechanism of action and experimental validation of key genes common to diabetic retinopathy and coronary heart disease based on multiple bioinformatics investigations

**DOI:** 10.3389/fgene.2025.1548147

**Published:** 2025-03-19

**Authors:** Fanli Jiang, Shi Yin, Xinjin Zhang

**Affiliations:** Department of Cardiology, The Affiliated Hospital of Yunnan University, Kunming, China

**Keywords:** diabetic retinopathy, coronary heart disease, bioinformatics, HIRIP3, ZNF416

## Abstract

**Introduction:**

The relationship between diabetic retinopathy (DR) and coronary artery disease (CHD) has been established as a reliable predictor. However, the underlying mechanisms linking these two conditions remain poorly understood. Identifying common key genes could provide new therapeutic targets for both diseases.

**Methods:**

Public databases were used to compile training and validation datasets for DR and CHD. Machine learning algorithms and expression validation were employed to identify these key genes. To investigate immune cell differences, single-sample gene set enrichment analysis (ssGSEA) and the Wilcoxon test were applied. Spearman correlation analysis further explored the relationship between key genes and immune cell variations. Additionally, potential therapeutic drugs targeting these key genes were identified and a key gene–drug network was constructed. The role of the key genes in the pathogenesis of DR and CHD was further examined through reverse transcription-quantitative polymerase chain reaction (RT-qPCR).

**Results:**

Consistent expression trends observed across datasets (GSE221521, GSE113079, GSE189005, GSE42148) led to the identification of *HIRIP3* and *ZNF416* as key genes. In GSE221521, HIRIP3 was positively correlated with CD56 bright natural killer cells (cor = 0.329, P < 0.001) and type 1T helper cells (cor = 0.327, P < 0.001), while *ZNF416* showed significant correlations with CD4 T cell activation (cor = 0.340, P < 0.001) and type 1T helper cells (cor = 0.273, P < 0.05). Moreover, 82 transcription factors (TFs) were predicted, including SP3. Binding free energy calculations for key genes and potential drugs suggested stable binding conformations. RT-qPCR results revealed elevated expression of both *HIRIP3* and *ZNF416* in the control group compared to the DR with CHD (DRwCHD) group, with only *ZNF416* showing significant differences between the groups (p < 0.05).

**Discussion:**

These findings highlight *HIRIP3* and *ZNF416* as crucial genes in DR and CHD detection, providing a foundation for identifying novel therapeutic targets for both diseases.

## 1 Introduction

Diabetic retinopathy (DR) represents a major complication of diabetes mellitus, posing substantial risks to both ocular health and overall wellbeing (Liu and Wu, 2021). As a leading global health concern, DR affects millions of individuals across the world (Tan and Wong, 2022). The pathogenesis of DR is predominantly driven by chronic hyperglycemia, which compromises the blood-retinal barrier’s integrity (Lin et al., 2021). This disruption results in leakage from the central retinal artery, ciliary vessels, and deeper retinal structures, leading to ischemic changes in the peripheral retina (Lin et al., 2021). In its advanced stages, DR can result in significant vision impairment, positioning it as one of the principal causes of blindness, especially among the aging population (Lin et al., 2021). Despite progress in clinical treatments, therapeutic options for DR remain limited by challenges such as low drug solubility, poor retinal permeability, potential toxicity to surrounding tissues, and rapid enzymatic degradation, leading to shortened therapeutic efficacy (Liu and Wu, 2021). As such, identifying reliable diagnostic genes linked to DR is critical for early intervention and prevention, offering potential to improve treatment strategies and reduce the societal burden of this debilitating disease.

Coronary heart disease (CHD) is a leading global health threat, contributing to high rates of morbidity and mortality (Shaya et al., 2022). A chronic immunoinflammatory and fibrotic condition, CHD is primarily driven by lipid accumulation and shaped by complex interactions of genetic, environmental, and lifestyle factors (Shaya et al., 2022). Despite significant advancements in surgical and pharmacological treatments, survival rates have improved but the post-myocardial infarction landscape is still fraught with complications and a marked decline in quality of life (Schwartz et al., 2018). Current interventions mainly involve pharmacotherapy and surgical revascularization. Although timely reperfusion and thrombolysis can alleviate adverse ventricular remodeling, these treatments do not fully restore myocardial structure, focusing more on symptom management rather than complete recovery (Chepeleva, 2023). Emerging evidence indicates that the presence of DR increases the risk of CHD, with patients with DR showing greater susceptibility to myocardial perfusion defects, diminished coronary flow reserve, and lower coronary collateral scores (Cheung et al., 2007).

Evidence indicates that DR not only affects visual function but may also negatively impact cardiovascular health. Multiple studies have demonstrated a significant association between DR and CHD. Specifically, diabetic patients with DR exhibit higher susceptibility to CHD development, potentially mediated through systemic inflammatory responses and endothelial dysfunction caused by microvascular damage (Zhang et al., 2024; Khazai et al., 2021). For instance, research revealed that type 2 diabetes patients with DR had significantly elevated CHD risk, even after adjusting for other known risk factors (Goldney et al., 2024). Mechanistic analyses suggest DR influences CHD progression through multiple pathways. First, DR serves as a biomarker for predicting macrovascular complications like CHD in diabetic patients. Second, both conditions share common risk factors such as hypertension and hypercholesterolemia, which exacerbate microvascular and macrovascular damage (Tanaka et al., 2021; Lin et al., 2024). In clinical practice, comprehensive evaluation of diabetic patients with DR is crucial for early identification of potential CHD risks and timely intervention. Further prospective studies are warranted to elucidate the intricate relationship between DR and CHD, aiming to develop more effective management strategies for this patient population.

This study utilized public databases and applied machine learning algorithms coupled with expression validation to identify shared key genes associated with both DR and CHD. Gene set enrichment analysis (GSEA) and gene set variation analysis (GSVA) were employed to explore the functional roles of these key genes, while immune infiltration analysis assessed their impact on immune responses within the context of both diseases. Additionally, regulatory networks were constructed to elucidate the molecular mechanisms underlying these key genes, and drug prediction models were developed to offer new clinical insights for the diagnosis and treatment of DR and CHD. The findings of this research underscore the interconnected pathways between these two diseases, providing a foundation for more targeted therapeutic strategies and, ultimately, improving patient outcomes in clinical practice.

## 2 Materials and methods

### 2.1 Acquisition of datasets

RNA sequencing data for this study were sourced from the Gene Expression Omnibus (GEO) database (https://www.ncbi.nlm.nih.gov/geo/), encompassing training set 1 (GSE221521, sequencing platform: GPL24676), training set 2 (GSE113079, sequencing platform: GPL20115), validation set 1 (GSE189005, sequencing platform: GPL23126), and validation set 2 (GSE42148, sequencing platform: GPL13607). The GSE221521 dataset included 50 control blood samples and 69 blood samples from patients with DR, while GSE189005 contained 9 control blood samples and 10 blood samples from patients with DR. The GSE113079 dataset comprised 48 control peripheral blood mononuclear cell (PBMC) samples and 93 PBMC samples from patients with CHD. The GSE42148 dataset included 11 control blood samples and 13 blood samples from patients with CHD.

### 2.2 Identification of potential candidate genes

To identify differentially expressed genes 1 (DEGs1), DESeq2 (v 1.38.0) (Badia-Bringué et al., 2024) was used to analyze GSE221521, resulting in the identification of DEGs1. The limma (v 3.54.0) package (Ritchie et al., 2015) was then applied to GSE113079 to detect DEGs2 (|log_2_fold change (log_2_FC)| > 0.5, adj.P.Val < 0.05) (Wang et al., 2022). A volcano plot for the top 15 DEGs with the greatest variation was generated using the ggplot2 (v 3.4.4) package (Aran et al., 2017), while a heatmap of the top 15 DEGs was visualized with ComplexHeatmap (v 2.14.0) (Gu and Hübschmann, 2022).

Subsequently, upregulated genes from DEGs1 and DEGs2 were intersected to form “intersecting gene 1,” and downregulated genes were intersected to form “intersecting gene 2.” These two sets were then combined to identify a set of potential candidate genes (PCGs) for further analysis. A Venn diagram, visualized using the VennDiagram (v 1.7.3) package (Zhou T. et al., 2023), was created to display the PCGs.

### 2.3 Enrichment and protein-protein interaction (PPI) network analysis of PCGs

To explore the cellular functions and pathways of the PCGs, Gene Ontology (GO) analysis was performed using ClusterProfiler (v 4.7.1.003) (Huang et al., 2024) and the human gene annotation package (org.Hs.eg.db) (v 3.16.0). A PPI network for the PCGs was constructed using Search Tool for the Retrieval of Interacting Genes/Proteins (STRING) (https://string-db.org) with a confidence score of 0.15, and network visualization was carried out using Cytoscape (v 3.8.2).

### 2.4 Access to key genes

To identify candidate genes, the area under the curve (AUC) values of PCGs were calculated using the pROC (v 1.18.0) package (Tsukita et al., 2023) in the GSE221521 and GSE113079 datasets. Genes with AUC values greater than 0.7 in both datasets were intersected, and the resulting genes were considered candidate genes for further analysis.

Subsequently, random forest classification models were developed using the randomForest (v 4.7-1.1) package (Cao et al., 2024) in both datasets to compute the Gini coefficient for each candidate gene. The Gini coefficient of these genes was compared, and the top 20 most important genes from GSE221521 and GSE113079 were selected. An intersection of the top 20 genes from both datasets revealed the hub genes. These hub genes were further analyzed using the support vector machines-recursive feature elimination (SVM-RFE) algorithm via the caret (v 6.0-93) package (Lv et al., 2024) in both datasets. Genes associated with the lowest error rates in the two datasets were identified as intersections and designated as signature genes for this study.

Subsequently, the Wilcoxon rank sum test was applied using the ggplot2 (v 3.4.4) package (Omar et al., 2024) to analyze the expression levels of signature genes across the GSE221521, GSE113079, GSE189005, and GSE42148 datasets (P < 0.05). The results were visualized using box-and-line plots. Genes that exhibited significant differences and consistent expression trends across these datasets were identified as key genes. The diagnostic value of these key genes for CHD and DR was further assessed using receiver operating characteristic (ROC) curves in the four datasets.

### 2.5 GSEA and GSVA of key genes

To explore the functional relevance of the key genes, the Molecular Signature Database (MSigDB) (https://www.gsea-msigdb.org/gsea/msigdb) was consulted, selecting “c5.go.bp.v7.4.symbols” as the reference gene set for functional analysis in GSE221521 and GSE113079. Spearman correlation analysis of the key genes with all other genes in these datasets was performed using the psych (v 3.4.4) package (Huang et al., 2020), and results were ranked based on the correlation coefficient. GSEA pathway enrichment was conducted using the ClusterProfiler (v 4.7.1.003) package (Wang et al., 2024), and the top 10 enriched pathways were selected for presentation (P.adjust < 0.05, |Normalized Enrichment Score (NES)| > 1).

Finally, disease samples were classified into high- and low-expression groups based on the median expression levels of the key genes. GSVA was performed using the GSVA (v 1.46.0) package, and significant pathway enrichment differences between the two groups were analyzed using the limma (v 3.54.0) package (Ritchie et al., 2015) with criteria of |t| > 2 and P < 0.05.

### 2.6 Immune microenvironment analysis

To explore immune microenvironment changes in DR and CHD, the single-sample GESA (ssGSEA) algorithm (Zhou J. et al., 2023) was employed to estimate the scores of 28 immune cell types (Zhang et al., 2023) across disease and control groups in GSE221521 and GSE113079. These immune cells were from the Tumor Immune System Interaction Database (TISIDB) (http://cis.hku.hk/TISIDB/). Differences in immune cell infiltration between the disease and control groups were assessed using the Wilcoxon test, with results visualized in box-and-whisker plots. This analysis allowed the identification of immune cell types exhibiting significant differences between disease and control groups, which were termed differential immune cells.

The role of key genes in the immune microenvironment was analyzed in the GSE221521 and GSE113079 datasets. To explore the potential relationships between key genes and differential immune cells, a Spearman correlation analysis was performed using the psych package (v 3.4.4) (Huang et al., 2020). The results were visualized in a lollipop diagram (P.adjust < 0.05).

### 2.7 Construction of key gene regulatory networks

Additionally, transcription factors (TFs) and microRNAs (miRNAs) are crucial in maintaining physiological stability by regulating target gene expression. To predict upstream TFs for the key genes, NetworkAnalyst (https://www.networkanalyst.ca/) was utilized to construct a TF-messenger RNA (TF-mRNA) regulatory network. miRNAs associated with key genes were predicted using the miRWalk database (http://mirwalk.umm.uni-heidelberg.de), and miRNA-mRNA regulatory networks were constructed.

### 2.8 Prediction of drug

To further investigate potential therapeutic agents targeting key genes, the Comparative Toxicogenomics Database (CTD) (https://ctdbase.org/) was consulted. A key genes-drug network was created and visualized using Cytoscape (v 3.8.2).

For drug-gene binding assessment, molecular docking of the key genes with core active drug ingredients was performed. The 3D structures of the drugs and protein molecular crystal structures of key genes were obtained from the National Center of Biotechnology Information (NCBI) PubChem Compound database (https://www.ncbi.nlm.nih.gov/pccompound/) and the Uniprot database (https://www.uniprot.org/), respectively. Among them, the protein structures of key genes were predicted by AlphaFold (https://alphafold.ebi.ac.uk/). The CB-Dock tool (https://cadd.labshare.cn/cb-dock/php/manual.php) was used to identify the optimal binding conformation of the protein and drug, with results visualized in PyMOL. The first step of CB-Dock was to predict the possible binding sites of the protein (Cavity detection). Since ligand - binding sites were usually some large cavities, several cavities with the highest scores were selected for further analysis according to the cavity size ranking (Cavity sorting). Subsequently, the center of the cavity needed to be set and the cavity size adjusted. These parameters were necessary for the molecular docking with AutoDock Vina (Center and Size). After docking, a series of binding poses were re - ranked according to the docking scores (Dock and Rerank). The first conformation was regarded as the best - binding conformation, and the corresponding site was the best - binding site of the query ligand. If the molecular binding free energy between the key gene and the drug was < −5.0 kcal/mol, it indicated a good docking affinity (Wu et al., 2022).

### 2.9 Reverse transcription-quantitative polymerase chain reaction (RT-qPCR)

This study included 10 fresh blood samples, comprising 5 samples from patients with comorbid diabetic retinopathy (DR) and coronary heart disease (CHD), along with 5 samples from healthy controls. All specimens were obtained from the Affiliated Hospital of Yunnan University. The research was approved by the institutional review board of the Affiliated Hospital of Yunnan University Ethics Committee (Approval No. 2024301), and all patients provided written informed consent. RNA extraction was performed using the TRIzol kit, with samples 1–5 designated as control and samples 6–10 as DRwCHD. All procedures for RNA extraction were conducted according to the manufacturer’s guidelines. The RNA concentration was assessed using 1 μL of extracted RNA and a NanoPhotometer N50, with the purity and concentration recorded to calculate the RNA required for subsequent reverse transcription. RNA was then reverse transcribed into cDNA using the SweScript First Strand cDNA Synthesis Kit, following the manufacturer’s instructions. The resulting DNA was diluted 5–20 times with RNase- and ARase-free ddH_2_O. A reaction mixture was prepared by adding 3 μL of cDNA, 5 μL of 2x Universal Blue SYBR Green qPCR Master Mix, 1 μL of forward primer (10 µM), and 1 μL of reverse primer (10 µM). The qPCR was performed for 40 cycles using the CFX96 real-time PCR system, with the detailed protocol provided in Supplementary Table S1. Primer sequences for *HIRIP3* and *ZNF416* are listed in Supplementary Table S2, with GAPDH serving as the reference gene. Relative gene expression levels were calculated using the 2^−ΔΔCT^ method.

### 2.10 Statistical analysis of data

All statistical analyses were conducted using R 4.2.3 software and the Cytoscape (v 3.8.2) platform. The Wilcoxon test was used to assess significant differences between groups, with a P-value < 0.05 considered statistically significant.

## 3 Results

### 3.1 Enrichment and PPI analysis in 96 potential candidate genes

**FIGURE 1:**
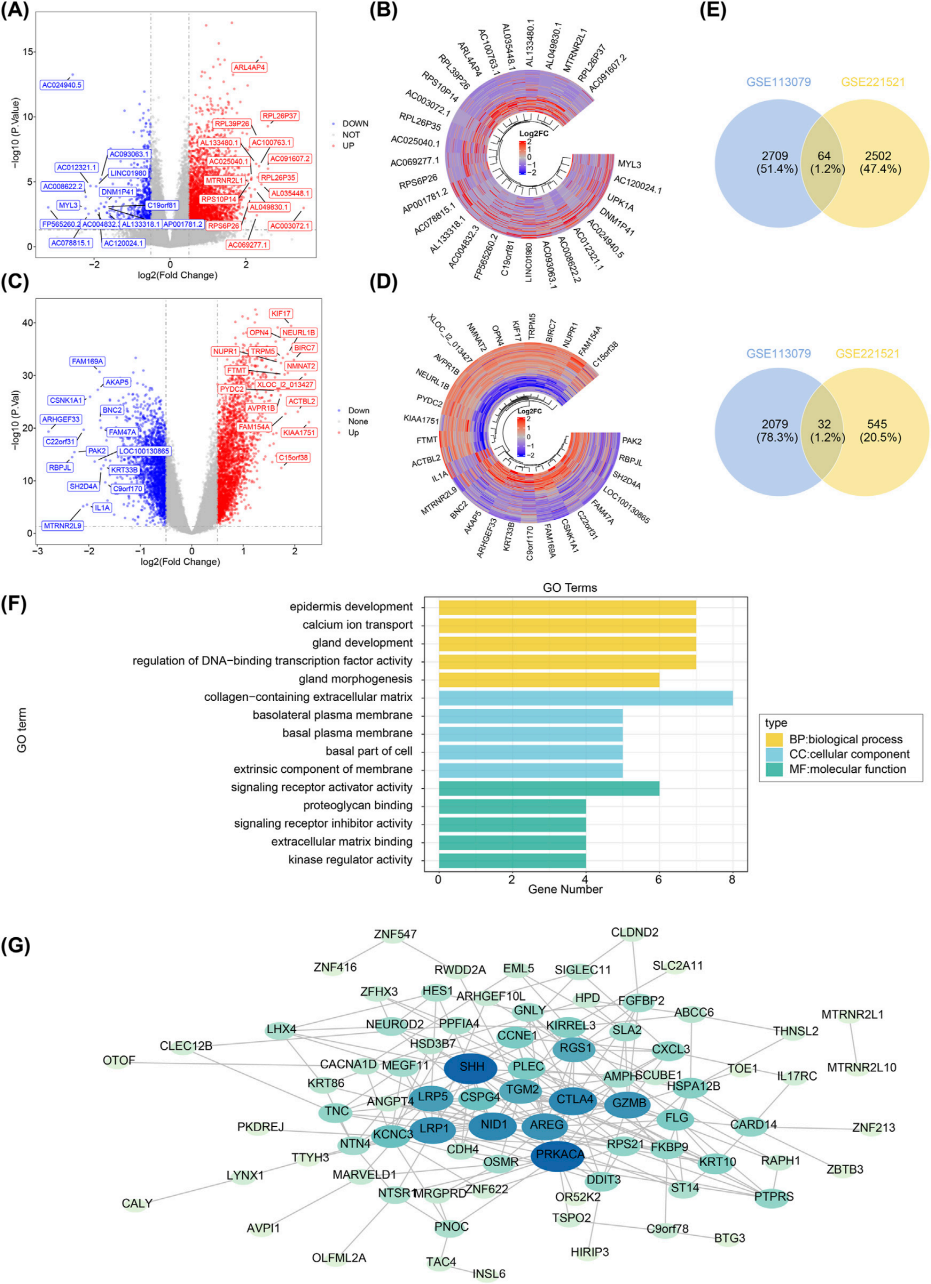
Screening of differentially expressed genes, functional enrichment analysis, and construction of protein-protein interaction networks. **(A)** Volcano plot showing differentially expressed genes in GSE221521. Grey dots represent genes with no significant expression change, blue dots indicate significantly downregulated genes, and red dots represent significantly upregulated genes. **(B)** Heatmap displaying the expression levels of the top 15 up- and downregulated differentially expressed genes in GSE221521. Yellow indicates high expression, and green represents low expression. **(C)** Volcano plot of differentially expressed genes in GSE113079. Grey dots represent genes with no significant expression change, blue dots indicate significantly downregulated genes, and red dots represent significantly upregulated genes. **(D)** Heatmap displaying the expression levels of the top 15 up- and downregulated differentially expressed genes in GSE113079. Yellow indicates high expression, and green represents low expression. **(E)** Venn diagram showing the intersection of upregulated genes between GSE221521 and GSE113079. **(F)** Venn diagram showing the intersection of downregulated genes between GSE221521 and GSE113079. **(G)** Gene ontology enrichment analysis of the co-aggregated genes. Protein-protein interaction network diagram.

A total of 3,143 DEGs1 were identified in GSE221521 (|log_2_FC| > 0.5, adj.P.Val < 0.05), including 2,566 upregulated and 577 downregulated genes in the DR group (Figures 1A, B). Similarly, 4,884 DEGs2 were identified in GSE113079 (|log_2_FC| > 0.5, adj.P.Val < 0.05), with 2,773 upregulated and 2,111 downregulated genes in the CHD group (Figures 1C, D). The results were visualized as a volcano map and heatmap. Using Venn analysis, 96 PCGs were identified for further functional enrichment analysis and PPI network construction (Figure 1E).

GO analysis revealed 207 biological processes (BPs), 32 cellular components (CCs), and 23 molecular functions (MFs) enriched by the 96 PCGs. Notably, in the BP category, the PCGs were primarily involved in epidermis development, calcium ion transport, and gland development. In terms of CC, PCGs were most enriched in the collagen-containing extracellular matrix. In the MF category, the PCGs were predominantly associated with signaling receptor activator activity (Figure 1F). Subsequently, a PPI network comprising 82 nodes and 217 edges was constructed (Figure 1G).

### 3.2 *HIRIP3* and *ZNF416* were identified as key genes

**FIGURE 2:**
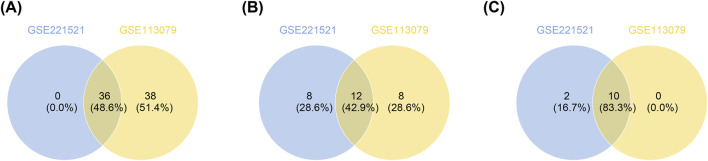
Screening of key genes by ROC analysis and machine learning. **(A)** Genes with AUC values greater than 0.7 in both training sets. **(B)** Venn diagram showing the intersection of the top 20 most important genes identified from the two training sets. **(C)** SVM-RFE models constructed for both training sets to identify genes corresponding to combinations with the lowest error rates. SVM-RFE: Support vector machine recursive feature elimination. AUC: Area under the curve; ROC: Receiver operating characteristic.

To identify key genes, ROC analysis revealed 36 candidate genes with an AUC > 0.7 in GSE221521 and GSE113079 (Figure 2A; Supplementary Table S3). Random forest analysis and Venn diagram analysis further identified 12 hub genes (ntree = 1,000), including *TOE1*, *OSMR*, *C9orf78*, *IL17RC*, *ZNF622*, *PRKACA*, *ZFHX3*, *CSPG4*, *ARHGEF10L*, *HIRIP3*, *PLEC*, and *ZNF416* (Supplementary Figure S1 and Figure 2B). Following this, 10 signature genes were identified through SVM-RFE analysis in GSE221521 and GSE113079 (Supplementary Figure S2 and Figure 2C). The Wilcoxon rank sum test revealed that *HIRIP3* and *ZNF416* were significantly differentially expressed with consistent expression patterns across GSE221521, GSE113079, GSE189005, and GSE42148 (P < 0.05). Notably, both genes showed higher expression in the control group across all datasets (Supplementary Figure S3), supporting their validation as reliable biomarkers. Additionally, ROC analysis demonstrated that both *HIRIP3* and *ZNF416* had strong diagnostic potential, with AUC values exceeding 0.7 (Supplementary Figure S4). Based on these findings, *HIRIP3* and *ZNF416* were identified as key genes for further exploration in this study.

### 3.3 GSEA and GSVA enrichment analyses in *HIRIP3* and *ZNF416*

**FIGURE 3:**
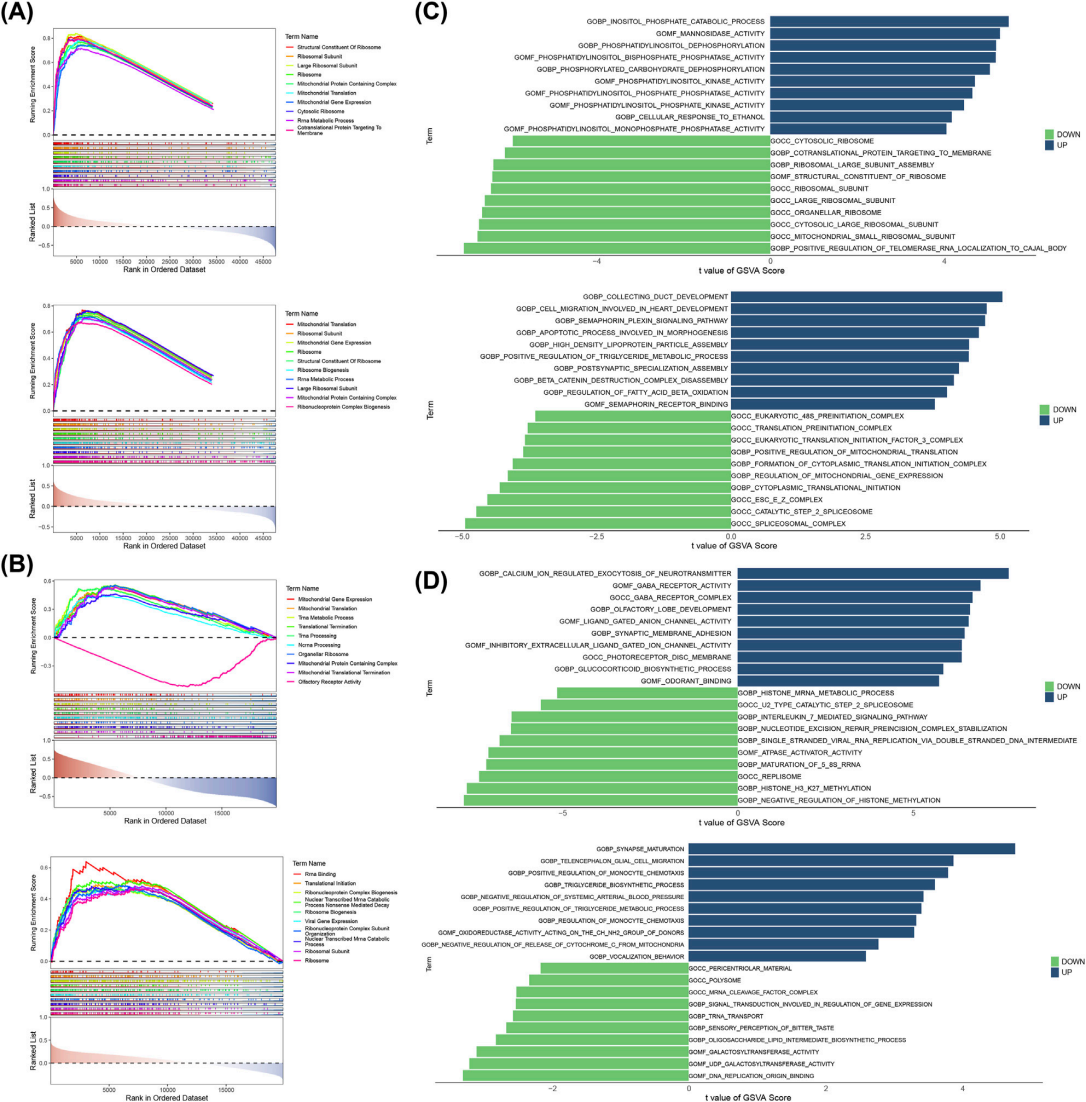
Results of GSEA and GSVA. **(A)** GSEA enrichment results for *HIRIP3* and *ZNF416* in the training set GSE221521. **(B)** GSEA enrichment results for *HIRIP3* and *ZNF416* in the training set GSE113079. **(C)** GSVA enrichment results for *HIRIP3* and *ZNF416* in the training set GSE221521. **(D)** GSVA enrichment results for *HIRIP3* and *ZNF416* in the training set GSE113079. GSEA: Gene set enrichment analysis; GSVA: Gene set variation analysis.

To investigate the functional phenotypes of key genes in GSE221521, GSEA enrichment analysis revealed that both *HIRIP3* and *ZNF416* were predominantly associated with the ribosomal subunit, ribosome, and rRNA metabolic processes (|NES| > 1, P.adjust < 0.05) (Figure 3A). In the GSE113079 dataset, *HIRIP3* was enriched in processes such as tRNA processing, ncRNA processing, and organellar ribosomes, while *ZNF416* was notably enriched in rRNA binding, translational initiation, and ribosomal functions (|NES| > 1, P.adjust < 0.05) (Figure 3B).

GSVA enrichment analysis of the two expression groups in GSE221521 indicated that *HIRIP3*-related upregulated pathways were primarily enriched in the inositol phosphate catabolic process, whereas downregulated *HIRIP3*-related pathways were linked to the positive regulation of telomerase RNA localization to the Cajal body. Conversely, *ZNF416*-related upregulated pathways were enriched in collecting duct development, and downregulated pathways were related to the spliceosomal complex (Figure 3C). In the GSE113079 dataset, *HIRIP3*-related upregulated pathways were primarily involved in calcium ion regulation of neurotransmitter release, while its downregulated pathways were associated with the negative regulation of histone methylation. *ZNF416*-related upregulated pathways were linked to synapse maturation, and downregulated pathways were enriched in DNA replication origin binding (Figure 3D).

### 3.4 Analysis of key genes with differential immunity cells

**FIGURE 4:**
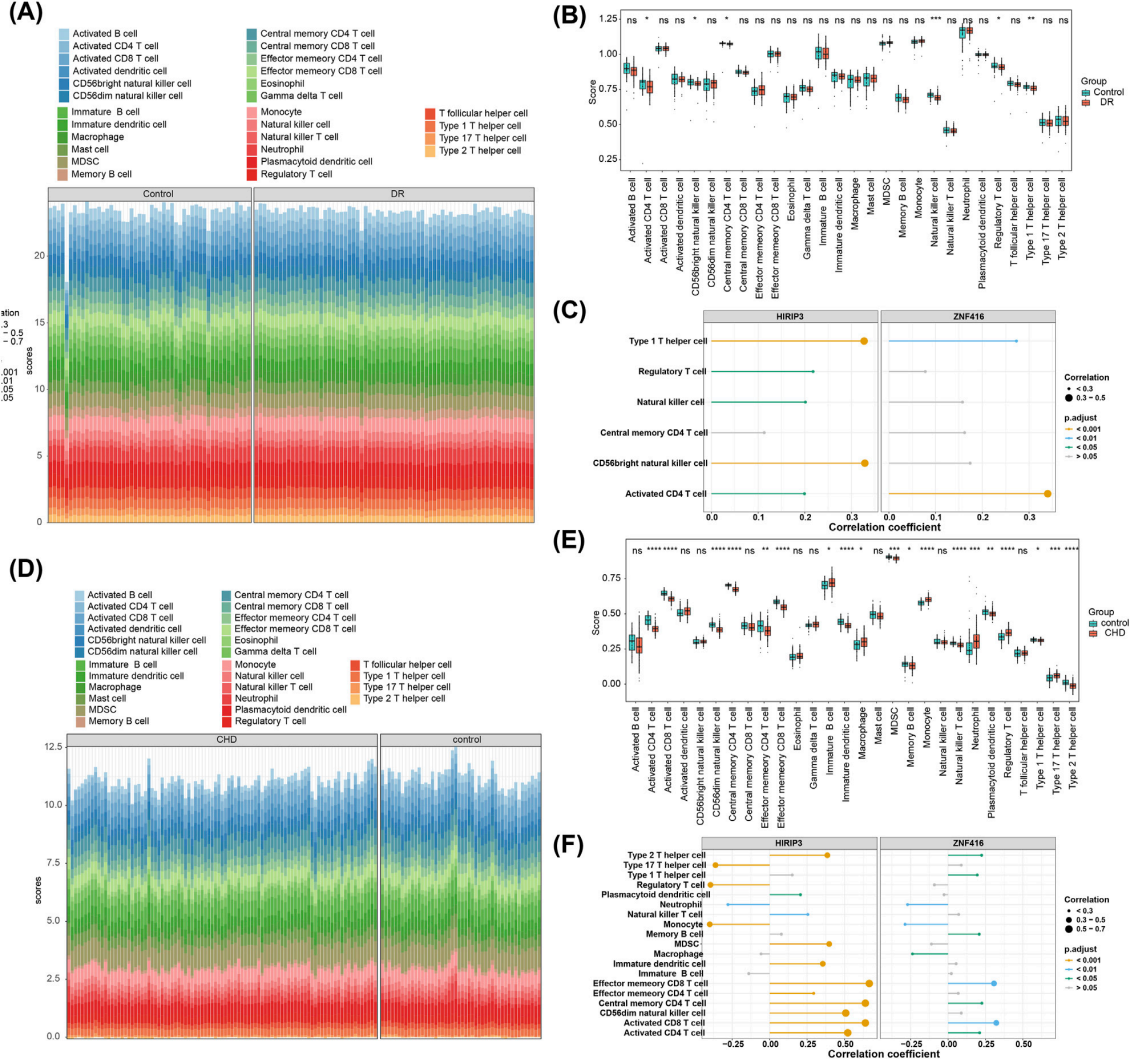
Immune infiltration analysis. **(A)** Stacked plot showing immune cell infiltration in the training set GSE221521. **(B)** Analysis of differential expression of immune cells in the disease and control groups in the training set GSE221521. **(C)** Correlation analysis results of *HIRIP3* and ZNF416 with differential immune cells in the training set GSE221521. **(D)** Stacked plot showing immune cell infiltration in the training set GSE113079. **(E)** Analysis of differential expression of immune cells in the disease and control groups in the training set GSE113079. **(F)** Correlation analysis results of *HIRIP3* and ZNF416 with differential immune cells in the training set GSE113079.

The objective of this study is to explore potential alterations in the immune microenvironment of individuals with DR and CHD. In the GSE221521 dataset, six differential immune cell types were identified, including CD4 T cell activation, CD56 bright natural killer cells, central memory CD4 T cells, natural killer cells, regulatory T cells, and type 1T helper cells (Figures 4A, B). Spearman correlation analysis revealed a positive correlation between *HIRIP3* and CD56 bright natural killer cells (cor = 0.329, adj.P < 0.001) as well as type 1T helper cells (cor = 0.327, adj.P < 0.001). *ZNF416* also showed a positive correlation with CD4 T cell activation (cor = 0.340, adj.P < 0.001) and type 1 T helper cells (cor = 0.273, adj.P < 0.05) (Figure 4C). In contrast, the GSE113079 dataset identified 19 differential immune cell types (Figures 4D, E). *HIRIP3* and *ZNF416* exhibited negative correlations with neutrophils (*HIRIP3*, cor = −0.279, adj.P < 0.001; *ZNF416*, cor = −0.270, adj.P < 0.05) and monocytes (*HIRIP3*, cor = −0.400; *ZNF416*, cor = −0.287) (adj.P < 0.001) (Figure 4F).

### 3.5 Constructing TF-mRNA and miRNA-mRNA networks to explore molecular regulatory mechanisms

**FIGURE 5:**
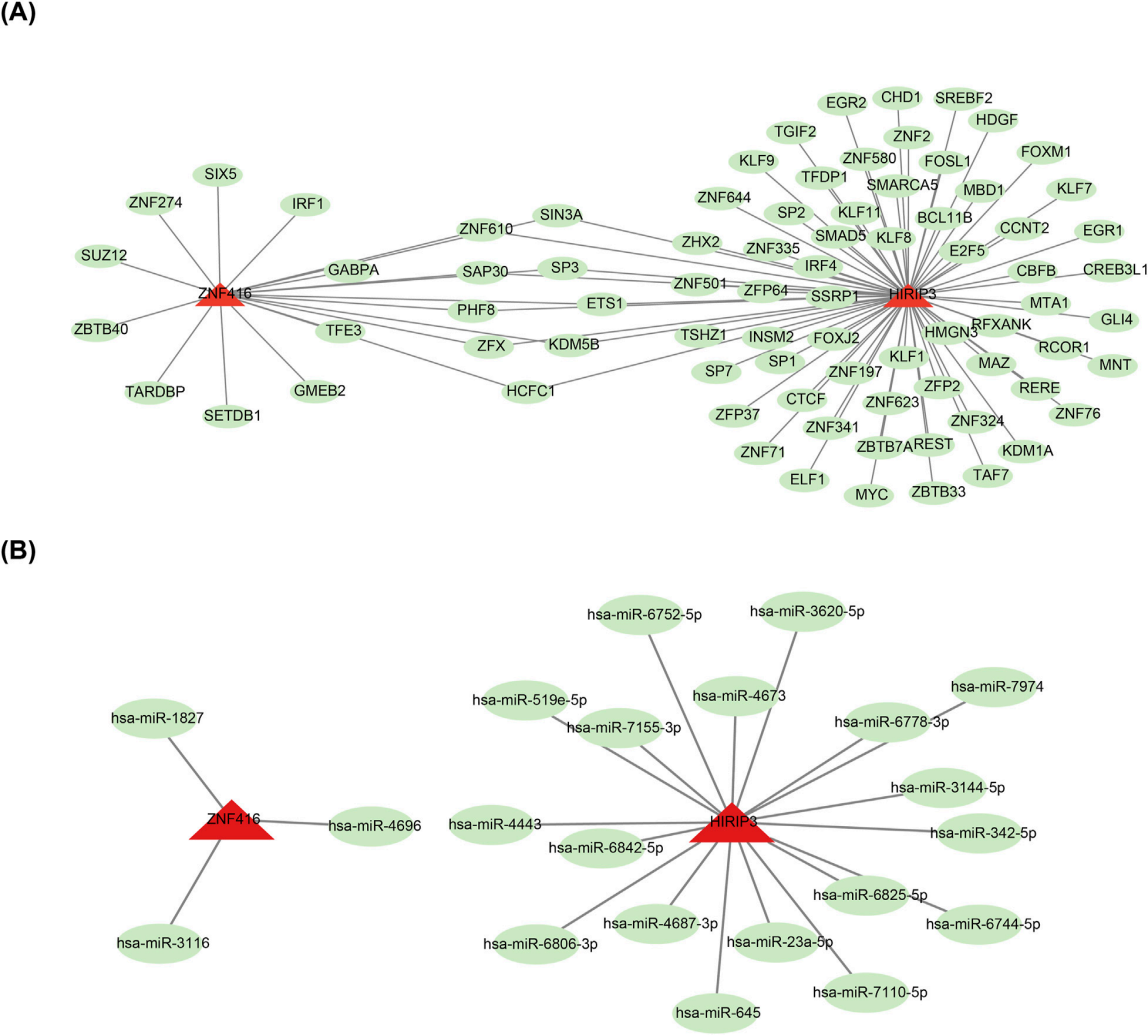
TF-mRNA and miRNA-mRNA regulatory networks. **(A)** Regulatory network of mRNA-miRNA. Red represents key genes (*HIRIP3*, *ZNF416*), and green represents TFs. **(B)** Regulatory network of miRNA-*HIRIP3*, ZNF416. Red represents key genes (*HIRIP3*, *ZNF416*), and green represents miRNAs. TF: Transcription factors.

To investigate the molecular regulatory mechanisms of *HIRIP3* and *ZNF416*, a total of 82 TFs, including SP3, were predicted for the two key genes (Figure 5A). Additionally, the miRNA-mRNA network analysis revealed that *HIRIP3* was associated with 18 predicted miRNAs, such as hsa-miR-645, while *ZNF416* was linked to only 3 miRNAs: hsa-miR-1827, hsa-miR-3116, and hsa-miR-4696 (Figure 5B).

### 3.6 Key genes and molecular docking

**FIGURE 6:**
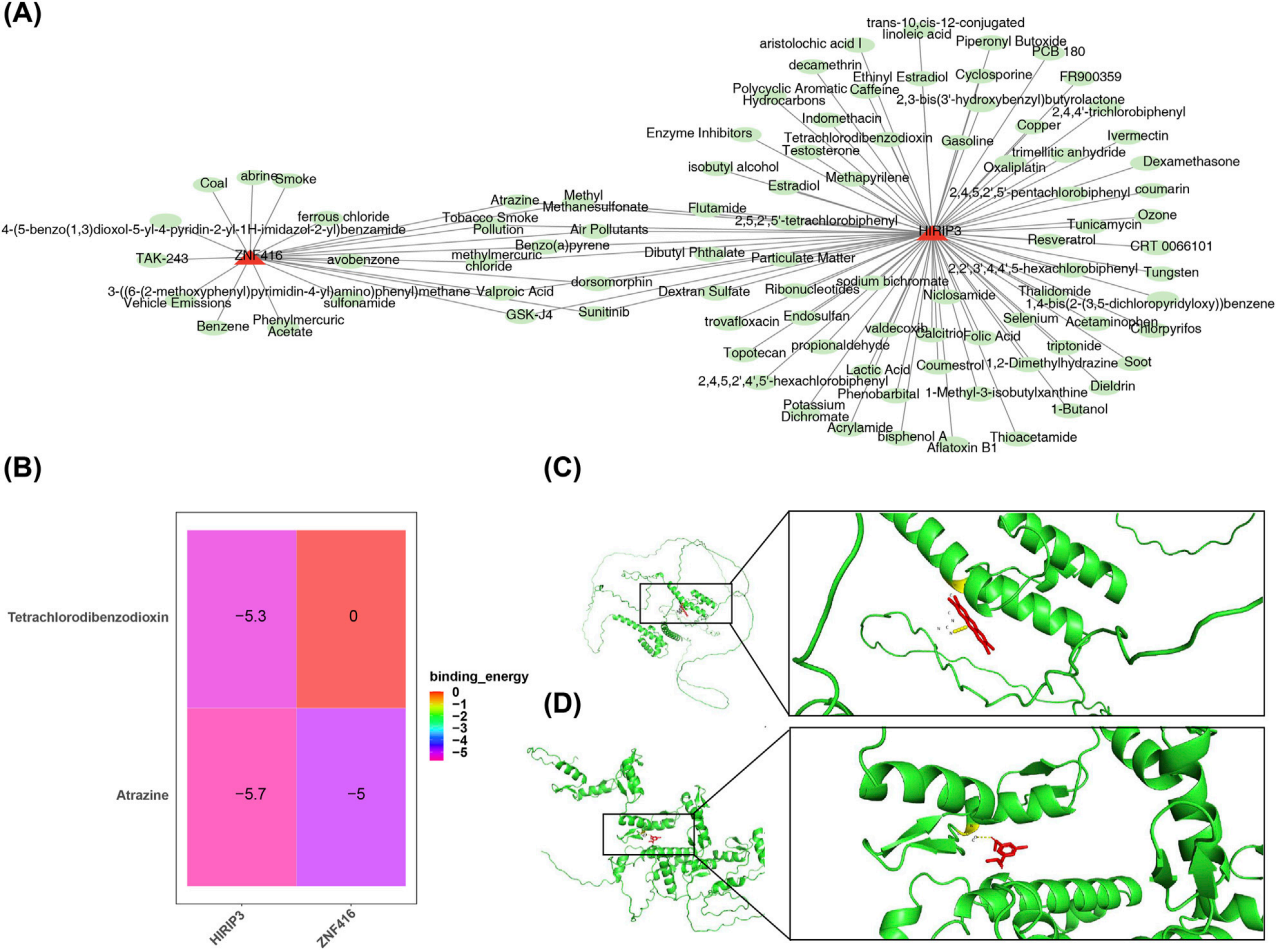
Drug prediction and molecular docking results. **(A)** Regulatory network of key gene-drug interactions. Red represents key genes (*HIRIP3*, *ZNF416*), green represents drugs. **(B)** Heatmap showing the binding results of molecular docking. **(C)** Molecular docking plot of HIRIP3 with Tetrachlorodibenzodioxin (−5.3 kcal/mol). **(D)** Molecular docking plot of ZNF416 with Atrazine (−5.0 kcal/mol).

Furthermore, 79 compounds were predicted for *HIRIP3*, and 20 for *ZNF416* (Figure 6A). Binding energy calculations showed that *HIRIP3* bound to Tetrachlorodibenzodioxin with a free energy of −5.3 kcal/mol, while *ZNF416* exhibited a binding energy of −5 kcal/mol with Atrazine (Figure 6B). These results suggest that the binding conformations of *HIRIP3* and *ZNF416* to Tetrachlorodibenzodioxin and Atrazine are stable, with the corresponding amino acid residues shown in Figures 6C, D.

### 3.7 Expression validation analysis

**FIGURE 7:**
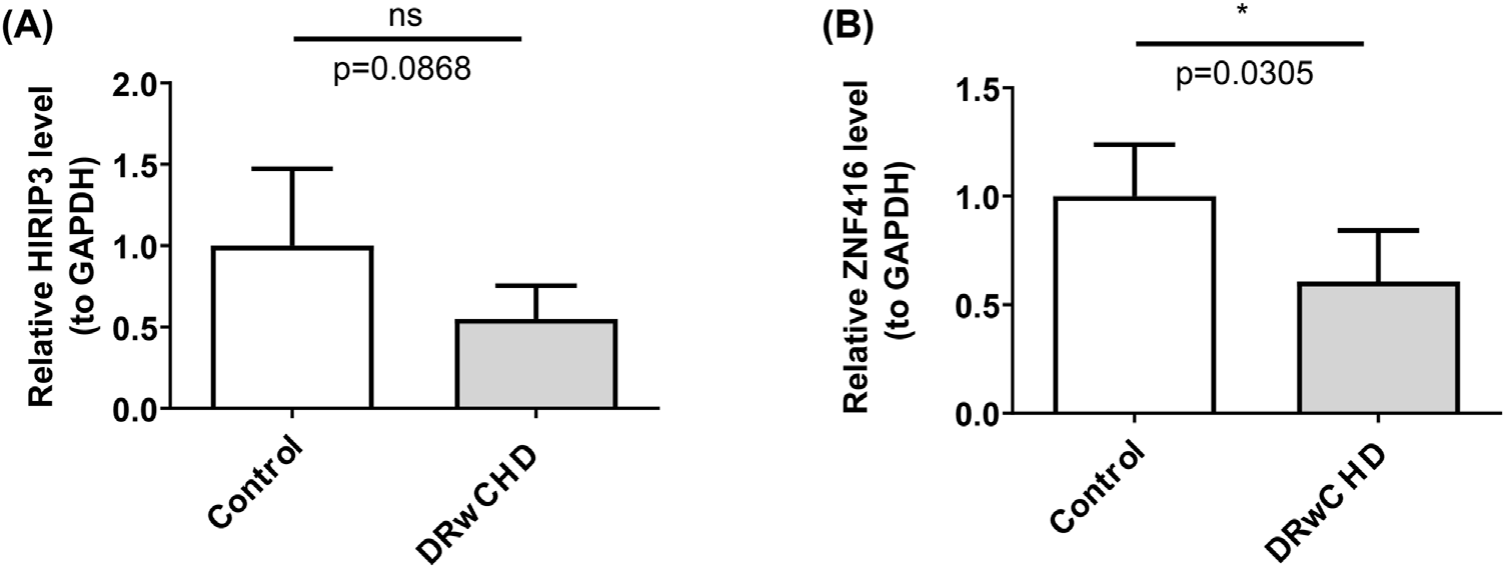
Expression validation analysis of key genes. **(A)** The expression level of *HIRIP3*. **(B)** [The expression level of *ZNF416*].

Expression validation analyses revealed significant differences in the expression levels of *HIRIP3* and *ZNF416* between the control and DRwCHD groups. Specifically, the expression of both genes was elevated in the control group compared to the DRwCHD group (Figures 7A, B). Notably, *ZNF416* showed a significant difference between the two groups (p < 0.05) (Figure 7B). These results underscore the pivotal roles of *HIRIP3* and *ZNF416* in understanding the pathogenesis of DR and CHD.

## 4 Discussion

DR and CHD present significant threats to human health, often leading to severe complications and elevated mortality. These conditions are interconnected, sharing common risk factors such as diabetes and cardiovascular disease, underscoring the necessity of understanding their shared molecular pathways. This study aimed to explore the biological functions, predictive potential, immune microenvironment, and possible drug candidates for key diagnostic genes common to both DR and CHD through bioinformatics approaches. Two pivotal genes, *HIRIP3* and *ZNF416*, were identified, which are implicated in the pathophysiology of both conditions, providing new insights into their molecular mechanisms.Notably, during preliminary validation of critical genes using RT-qPCR, HIRIP3 and ZNF416 exhibited significantly higher expression levels in the control group than in the DRwCHD group. However, only ZNF416 exhibited a statistically significant difference (p < 0.05). The lack of a significant difference in *HIRIP3* expression could be attributed to the limited sample size, which restricted the study’s power to detect true differences. To address this, future research will involve larger sample sizes or alternative validation strategies using diverse sample types, allowing for a more robust assessment of *HIRIP3*’s role in disease progression. Increasing the sample size will provide a more accurate reflection of population heterogeneity and offer a deeper understanding of *HIRIP3*’s involvement in DR and CHD. These findings not only advance our understanding of the molecular interplay between DR and CHD but also highlight the potential of these genes as clinical biomarkers and therapeutic targets, ultimately aiming to improve patient outcomes in both conditions.

*ZNF416*, a zinc finger nuclease, is implicated in fibroblast activation, playing a pivotal role in fibroblast proliferation, extracellular matrix (ECM) synthesis, and contractility (Huang et al., 2020). Fibroblasts are central to tissue homeostasis and repair, primarily through the synthesis of ECM components (Cheng et al., 2022). ECM remodeling is essential for the progression of proliferative diabetic retinopathy (PDR) (Korhonen et al., 2021) and is also a key factor in coronary artery disease, where the degradation of ECM components such as collagen, elastin, and proteoglycans is associated with inflammatory responses. Several ECM degradation markers have been linked to the presence, severity, and prognosis of coronary artery disease (Theofilis et al., 2022)In DR, fibrosis primarily manifests as abnormal thickening of retinal vascular structures and neovascularization. These pathological changes increase vascular permeability, triggering retinal edema and hemorrhage, which may ultimately lead to vision loss (Klaassen et al., 2015). Meanwhile, in CHD, progressive fibrosis drives gradual accumulation of extracellular matrix (ECM) between cardiomyocytes, resulting in impaired cardiac diastolic and systolic functions (Perreault et al., 2024; Meng et al., 2024). Emerging evidence from in-depth studies on ZNF416 function reveals its critical role not only in pulmonary fibrosis but also potentially in other fibrotic diseases. However, the precise mechanisms of ZNF416 action remain unclear, particularly regarding its functional variations across different fibrotic conditions. ZNF416 plays a pivotal role in modulating gene expression, cell proliferation, and apoptosis, and likely impacts the progression of diabetes-related complications through its regulation of cellular metabolic processes and inflammatory responses (Chu et al., 2022). Our experimental validation results demonstrate that the low expression of ZNF416 in the disease group suggested its potential role as a protective factor in diabetic retinopathy complicated with coronary heart disease. Further investigation into *ZNF416*s role in the molecular mechanisms of DR and CHD is essential, as it may lead to the development of novel therapeutic strategies for managing these prevalent comorbid conditions.

Previous research has identified *HIRIP3* as a candidate gene associated with aortic valve stenosis (Ghebranious et al., 2007). The *HIRIP3* gene product is thought to interact with histone cell cycle regulator and core histones H2B/H3, suggesting its involvement in chromatin dynamics and histone metabolism, which are critical for cardiac development (Jones et al., 2021). Research involving fetuses with recurrent microdeletions at the 16p11.2 locus has shown that the second *HIRIP3* allele lacks additional mutations, implying that haploinsufficiency of *HIRIP3* may contribute to cardiovascular malformations (Ignatyeva et al., 2024). These findings highlight *HIRIP3*’s significant role in various cardiac conditions. However, its association with DR remains underexplored. Given the intertwined nature of cardiovascular health and diabetes-related complications, further investigation into *HIRIP3*’s role in DR and CHD is essential. This study is the first to report the association of *HIRIP3* with both CHD and DR. *HIRIP3* emerges as a novel therapeutic target, and understanding its contribution to these diseases could unveil shared molecular pathways, thereby facilitating the development of targeted therapies to mitigate the risks associated with diabetes and its complications.

GSEA results in this study revealed that key genes are co-enriched in the ribosome pathway in both diseases. Ribosomes are large RNA-protein complexes responsible for translating nucleic acid sequences into proteins, which are primary biochemical components of cells (Nofal and Rabinowitz, 2018). These structures are essential for cell growth, and any disruption in protein synthesis, particularly in the heart, can impair cardiomyocyte function, potentially contributing to the development of CHD (Nofal and Rabinowitz, 2018). Additionally, the dysregulation of ribonucleoproteins, which govern ribosomal activity, due to exposure to saturated fatty acids, plays a significant role in diabetic cardiac vulnerability to ischemia/reperfusion injury (Zhao et al., 2017). Furthermore, mitochondrial ribosomal protein L7/L12 (MRPL12) has been suggested to compensate for diabetic ischemic heart disease, indicating its potential involvement in diabetic myocardial infarction (Rai et al., 2024). Notably, mitochondrial ribosomal deficiencies in β cells are linked to type 2 diabetes-associated islet failure (Hong et al., 2022). In summary, ribosomes are critical in both cardiac and diabetic processes. While no direct studies have connected ribosomes to DR and CHD, DR is a major complication of diabetes, and CHD is a leading cause of heart disease. Therefore, it is hypothesized that the key genes identified in this study may influence the onset and progression of both CHD and DR by regulating the ribosome pathway.

The key genes jointly predicted three compounds: Atrazine, valproic acid, and sunitinib. Atrazine has been implicated in exacerbating myocardial fibrosis by inducing cardiomyocyte pyroptosis, highlighting its potential role in the progression of cardiac fibrosis (Zhao et al., 2024). Additionally, studies have reported retinal degeneration, including the degeneration of cone and rod photoreceptors, as a consequence of atrazine exposure (Ghisolfi et al., 1983). Given these effects, atrazine is considered detrimental to both DR and CHD, and its exposure should be minimized or avoided where possible.

Molecular docking was a method to predict the binding modes and affinities of two or more molecules by simulating intermolecular interactions. Binding energy was an important indicator for measuring the binding strength between molecules, which reflected the stability of the binding between the ligand and the receptor. If the binding energy was high, it indicated that the ligand and the receptor bound tightly and might have good biological activity. Conversely, if the binding energy was low, the binding between the ligand and the receptor might be unstable and the biological activity might be weak (Wu et al., 2022; Naqvi et al., 2018). Valproic acid has demonstrated significant therapeutic potential, reducing plasma glucose levels, HbA1c, insulin resistance, and fat accumulation in brown and white adipose tissue, as well as in the liver, with effects comparable to metformin treatment (Khan et al., 2016). Magnesium valproate has been shown to halt disease progression in the early stages of diabetic cardiomyopathy, potentially through the upregulation of estrogen receptors in left ventricle (LV) tissue (Rabadiya et al., 2018). Consequently, valproic acid holds promise as a therapeutic agent for both DR and coronary heart disease. Sunitinib, however, has cardiac toxicities, including hypertension, left ventricular ejection fraction (LVEF) dysfunction, congestive heart failure (CHF), and arterial thromboembolism (Pantaleo et al., 2012). In contrast, sunitinib has demonstrated inhibitory effects on choroidal neovascularization (CNV), a vision-threatening condition common in the elderly, in a laser-induced CNV mouse model, showing its potential as a therapeutic agent (Tavakoli et al., 2022). Thus, sunitinib may have divergent effects in the treatment of DR and CHD.

In this study, both key genes exhibited a significant positive correlation with activated CD4 T cells across the two training cohorts. Previous studies have suggested that T follicular helper (Tfh) cells, a recently identified subset of CD4^+^ T cells, play a role in retinal vasculitis associated with DR. Tfh cells, directed by Bcl-6, can promote vascular inflammation and angiogenesis, providing new avenues for DR treatment (Liu et al., 2020). Interestingly, the proportion of CD4 (+)CD25 (+) regulatory T cells (Tregs) in patients with CHD is significantly lower compared to controls, indicating a strong link between CD4 T cells and CHD. Alterations in these cell populations may lead to reduced peripheral autoimmune tolerance, contributing to the onset and progression of CHD (Zhao et al., 2007). Thus, it is hypothesized that the key genes *HIRIP3* and *ZNF416* may influence the development of both DR and CHD by modulating CD4 T cell infiltration.

This study utilized bioinformatics approaches to identify shared key genes and molecular processes between DR and CHD, facilitating drug prediction based on these genes. By elucidating the mechanisms underlying the pathogenesis of these prevalent conditions, our research provides a foundation for improving clinical diagnosis and treatment strategies for patients with both DR and CHD. However, we fully acknowledge that the relatively small sample size due to constrained experimental funding and tight research timelines may limit statistical power, potentially preventing the detection of certain effects. Therefore, future studies will involve larger-scale cohorts to enhance statistical robustness and validate our findings. While current expression differences and correlational analyses partially support the potential roles of *HIRIP3* and *ZNF416* genes in DR and CHD, these findings primarily rely on data correlations and lack direct functional validation. Subsequent investigations will incorporate functional experiments such as gene knockout/overexpression studies and animal models mimicking human disease states to verify the biological significance of our observations. Further research is essential to validate the relevance of these key genes in clinical practice, thereby advancing the understanding of the interplay between DR and CHD and contributing to improved patient outcomes through ongoing research in this vital area. Addressing these challenges will refine the insights gained from this study and translate them into tangible clinical benefits.

## Data Availability

The original contributions presented in the study are included in the article/Supplementary Material, further inquiries can be directed to the corresponding author.

## Abbreviations

DR, Diabetic retinopathy; DEGs, Differentially expressed genes; SVM-RFE, Support vector machine recursive feature elimination; FC, Fold change; LASSO, Least absolute shrinkage and selection operator; Laryngeal cancer; RT-qPCR, Reverse transcription-quantitative polymerase chain reaction; Principal component analysis; GEO, Gene expression omnibus; GO, Gene ontology; KEGG, Kyoto encyclopedia of genes and genomes; AUC, Area under curve; BP, Biological process; MF, Molecular function; CC, Cellular component; ROC, Receiver operating characteristic; ssGSEA, Single-sample gene set enrichment analysis; TFs, Transcription factors; GSVA, Gene set variation analysis; CHD, Coronary heart disease; PCGs, Potential candidate genes; mRNA, Messenger RNA; PPI, Protein-protein interaction.
