# Supplementary material for: Mechanism of action and experimental validation of key genes common to diabetic retinopathy and coronary heart disease based on multiple bioinformatics investigations
Source: Front Genet. 2025 Mar 19;16:1548147. doi: 10.3389/fgene.2025.1548147 (PMC11961877; doi:10.3389/fgene.2025.1548147)
Supplement: Supplementary file 1 [file DataSheet1.pdf]

Supplementary Material

1 Supplementary Figures and Tables

1.1 Supplementary Figures

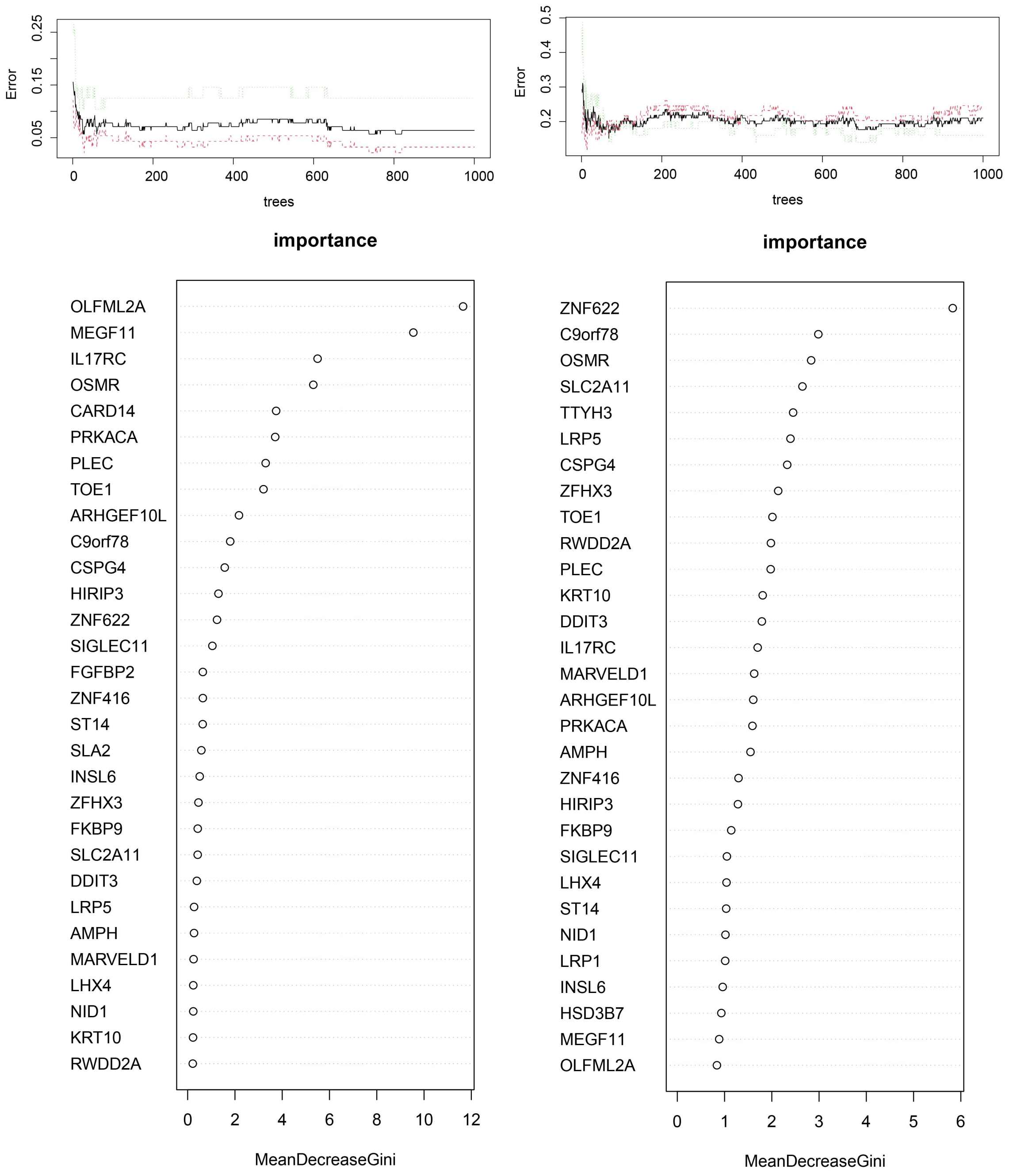

Supplementary Figure 1. Decision trees for RF model in training set GSE221521 and GSE113079. The horizontal coordinate was the number of decision trees, and the vertical coordinate was the model error rate. RF: Random forest.

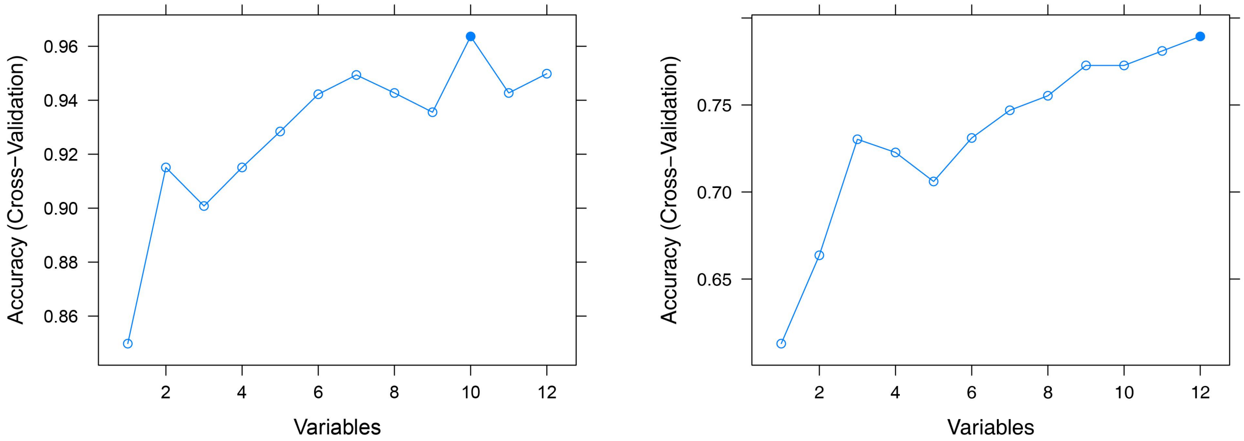

Supplementary Figure 2. Results of SVM-RFE analysis in training set GSE221521 and GSE113079. The horizontal coordinate was the number of feature genes, and the vertical coordinate was the model prediction accuracy. SVM-RFE: Support vector machine recursive feature elimination.

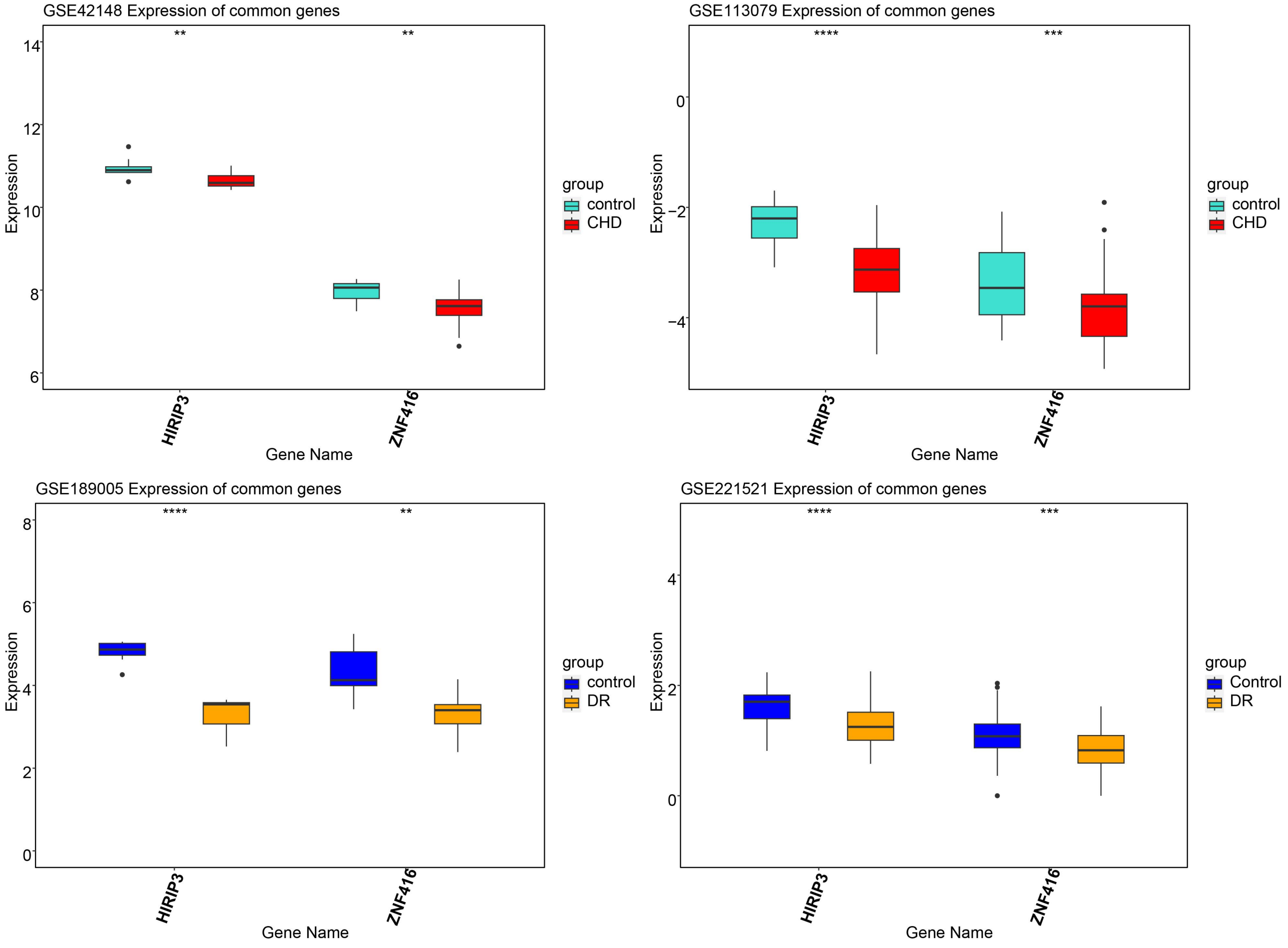

Supplementary Figure 3. Differences in expression levels of key genes (HIRIP, ZNF416) in the training set (GSE221521, GSE113079) and validation set (GSE189005, GSE42148).

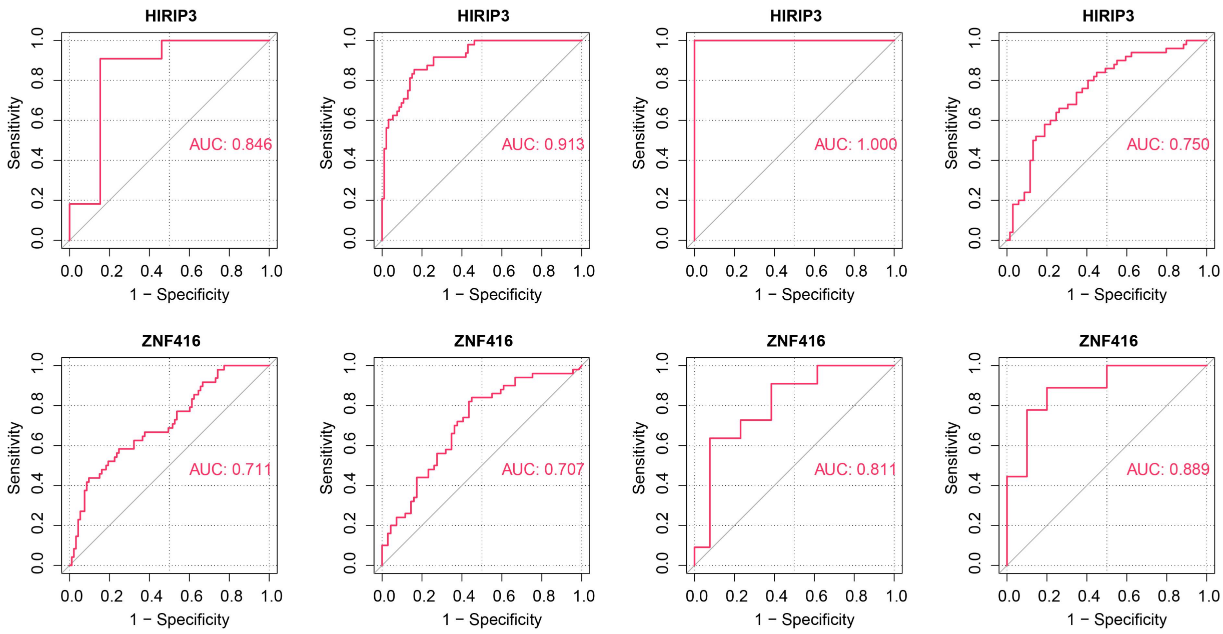

Supplementary Figure 4. Plotting ROC curves for key genes (*HIRIP*, *ZNF416*) in datasets GSE42148, GSE113079, GSE189005, and GSE221521. ROC: Receiver operating characteristic.

1.2 Supplementary Tables

Supplementary Table 1. The procedure information.

	Temperature	Time
Initial denaturation	95℃	1min
Denaturation	95℃	20s
Annealing	55℃	20s
Txtension	72℃	30s

Supplementary Table 2. The primer sequences.

Frimer	Sequence
HIRIP3 F	AGATGCAGGTGGATGAAGCC
HIRIP3 R	CATTCTTTGCTGGGGGTCCA
ZNF416 F	GATTCGACTTCGGTTCCCGT
ZNF416 R	CCAACAAACCAGCGCAGTTA
GAPDH F	CGAAGGTGGAGTCAACGGATTT
GAPDH R	ATGGGTGGAATCATATTGGAAC

Supplementary Table 3. ROC curves of 36 candidate genes in GSE221521 and GSE113079 sets.

GSE113079			GSE221521		
	Gene	AUC		Gene	AUC
1	ABCC6	0.95	6	AMPH	0.736
2	KIRREL3	0.888	7	SLA2	0.713
3	HSPA12B	0.916	8	PRKACA	0.713
4	THNSL2	0.949	10	PLEC	0.715
5	HPD	0.724	11	TGM2	0.703
6	AMPH	0.811	13	NID1	0.735
7	SLA2	0.811	18	KRT10	0.778
8	PRKACA	0.891	19	OSMR	0.797
9	RAPH1	0.93	20	ST14	0.717

10	PLEC	0.922	21	LRP5	0.709
11	TGM2	0.786	28	ARHGEF10L	0.736
12	ANGPT4	0.792	29	LRP1	0.785
13	NID1	0.733	30	SIGLEC11	0.707
16	GZMB	0.914	32	MARVELD1	0.777
17	CCNE1	0.731	34	C9orf78	0.784
18	KRT10	0.934	36	FKBP9	0.72
19	OSMR	0.973	43	CARD14	0.709
20	ST14	0.722	44	IL17RC	0.752
21	LRP5	0.784	46	ZNF213	0.735
23	SHH	0.924	48	MEGF11	0.705
25	FLG	0.913	50	CSPG4	0.771
26	GNLY	0.871	53	FGFBP2	0.701
27	CTLA4	0.839	58	DDIT3	0.722
28	ARHGEF10L	0.877	59	HIRIP3	0.75
29	LRP1	0.768	62	TOE1	0.807
30	SIGLEC11	0.871	63	LHX4	0.701
31	AVPI1	0.839	64	HSD3B7	0.706
32	MARVELD1	0.877	66	INSL6	0.726
33	BTG3	0.835	67	TAC4	0.719

34	C9orf78	0.917	70	ZNF622	0.816
35	TSPO2	0.963	71	SLC2A11	0.72
36	FKBP9	0.801	73	TTYH3	0.764
37	CACNA1D	0.776	77	ZFHX3	0.741
38	OTOF	0.871	78	OLFML2A	0.717
39	EML5	0.898	80	RWDD2A	0.726
40	KCNC3	0.917	82	ZNF416	0.707
41	CALY	0.709			
42	LYNX1	0.83			
43	CARD14	0.951			
44	IL17RC	0.973			
45	SCUBE1	0.887			
46	ZNF213	0.858			
47	ZBTB3	0.835			
48	MEGF11	0.98			
49	CDH4	0.869			
50	CSPG4	0.885			
51	PTPRS	0.934			
52	CLDND2	0.824			
53	FGFBP2	0.815			
54	CLEC12B	0.787			

55	TNC	0.89
56	PPFIA4	0.947
57	NEUROD2	0.792
58	DDIT3	0.752
59	HIRIP3	0.913
60	RPS21	0.802
62	TOE1	0.927
63	LHX4	0.728
64	HSD3B7	0.779
66	INSL6	0.803
67	TAC4	0.818
68	PKDREJ	0.847
70	ZNF622	0.907
71	SLC2A11	0.882
73	TTYH3	0.775
74	MRGPRD	0.919
75	MTRNR2L1	0.801
76	MTRNR2L10	0.891
77	ZFHX3	0.757
78	OLFML2A	0.949

79	OR52K2	0.821
80	RWDD2A	0.799
81	ZNF547	0.794
82	ZNF416	0.711
